# Genetic Dissection of Grain Nutritional Traits and Leaf Blight Resistance in Rice

**DOI:** 10.3390/genes10010030

**Published:** 2019-01-08

**Authors:** Gwen Iris Descalsota-Empleo, Abd Aziz Shamsudin Noraziyah, Ian Paul Navea, Chongtae Chung, Maria Stefanie Dwiyanti, Reuben Jacob Dicen Labios, Asmuni Mohd Ikmal, Venice Margarette Juanillas, Mary Ann Inabangan-Asilo, Amery Amparado, Russell Reinke, Casiana M. Vera Cruz, Joong Hyoun Chin, B.P. Mallikarjuna Swamy

**Affiliations:** 1 International Rice Research Institute (IRRI), Laguna 4031, Philippines; gidescalsota@gmail.com (G.I.D.-E.); ipnavea@gmail.com (I.P.N.); dwiyanti@abs.agr.hokudai.ac.jp (M.S.D.); jacob.labios@gmail.com (R.J.D.L.); v.juanillas@irri.org (V.M.J.); m.inabangan@irri.org (M.A.I.-A.); a.amparado@irri.org (A.A.); r.reinke@irri.org (R.R.); c.veracruz@irri.org (C.M.V.C.); 2 University of the Southern Mindanao, Kabacan, Cotabato 9407, Philippines; 3 Faculty of Science and Technology, Universiti Kebangsaan Malaysia, 43600 Bangi, Selangor, Malaysia; nora_aziz@ukm.edu.my (A.A.S.N.); mohdikmal@siswa.ukm.edu.my (A.M.I.); 4 Nousbo Corp. #4-107, 89 Seohoro, Gwonsun, Suwon 16614, Gyeonggi, Korea; 5 Chungcheongnam-do Agricultural Research and Extension Services, 167, Chusa-ro, Shinam-myeon, Yesan-gun 32418, Chungcheongnam-do, Korea; chts6991@korea.kr; 6 Applied Plant Genome Laboratory, Hokkaido University, Kita 9, Nishi 9, Kita-ku, Sapporo 060-8589, Japan; 7 Department of Integrative Bio-Industrial Engineering, Sejong University, 209, Neungdong-ro, Gwangjin-gu, Seoul 05006, Korea

**Keywords:** colored rice, nutritional content, Fe, Zn, anthocyanin, bacterial leaf blight, genome-wide association mapping, QTLs

## Abstract

Colored rice is rich in nutrition and also a good source of valuable genes/quantitative trait loci (QTL) for nutrition, grain quality, and pest and disease resistance traits for use in rice breeding. Genome-wide association analysis using high-density single nucleotide polymorphism (SNP) is useful in precisely detecting QTLs and genes. We carried out genome-wide association analysis in 152 colored rice accessions, using 22,112 SNPs to map QTLs for nutritional, agronomic, and bacterial leaf blight (BLB) resistance traits. Wide variations and normal frequency distributions were observed for most of the traits except anthocyanin content and BLB resistance. The structural and principal component analysis revealed two subgroups. The linkage disequilibrium (LD) analysis showed 74.3% of the marker pairs in complete LD, with an average LD distance of 1000 kb and, interestingly, 36% of the LD pairs were less than 5 Kb, indicating high recombination in the panel. In total, 57 QTLs were identified for ten traits at *p* < 0.0001, and the phenotypic variance explained (PVE) by these QTLs varied from 9% to 18%. Interestingly, 30 (53%) QTLs were co-located with known or functionally-related genes. Some of the important candidate genes for grain Zinc (Zn) and BLB resistance were *OsHMA9, OsMAPK6, OsNRAMP7, OsMADS13,* and *OsZFP252,* and *Xa1, Xa3, xa5, xa13* and *xa26,* respectively. Red rice genotype, Sayllebon, which is high in both Zn and anthocyanin content, could be a valuable material for a breeding program for nutritious rice. Overall, the QTLs identified in our study can be used for QTL pyramiding as well as genomic selection. Some of the novel QTLs can be further validated by fine mapping and functional characterization. The results show that pigmented rice is a valuable resource for mineral elements and antioxidant compounds; it can also provide novel alleles for disease resistance as well as for yield component traits. Therefore, large opportunities exist to further explore and exploit more colored rice accessions for use in breeding.

## 1. Introduction

Rice, being a dominant cereal and staple food, provides energy and nutrition for a majority of the Asian population [1]. In general, white rice is consumed as a major part of the daily diet, which is low in nutritional value in comparison with brown rice or pigmented rice [2]. However, because of increased health consciousness in recent years, there has been a greater awareness among the general population of the health and nutritional benefits of brown rice or pigmented rice. As a result, demand has increased for colored rice and its by-products from food, health, and cosmetic industries, which has created market and export opportunities for the major rice-growing countries in Asia [3,4,5,6].

It is well known that colored rice is rich in proteins, vitamins, minerals, fiber, and also phytochemicals such as tocopherols, tocotrienols, δ-oryzanols, phenolic compounds, etc., which offer several health and nutritional benefits [7,8,9,10]. Hence, many efforts have been made to promote brown rice and traditional rice varieties and also to enrich the nutritional value of modern rice varieties [11,12,13]. Among the different elements, Fe and Zn and anthocyanin-related antioxidant compounds are essential for normal growth and development and leading a healthy life [7,8]. It has been reported that more than one-third of the human population globally is affected by Fe and Zn deficiency, malnutrition, and oxidative stress-related health problems [14,15]. Fe deficiency causes anemia and poor immunity and cognitive development, along with higher maternal and pre-natal mortalities [16,17,18]. Zn deficiency results in diarrhea, stunting, poor cognitive development, low fertility, anorexia, etc. [17]; whereas oxidative stress causes several health problems such as stroke, psoriasis, dermatitis, and rheumatoid arthritis [19]. These health and nutritional problems have to be addressed urgently in order to achieve sustainable development goals by reducing the mortality of children and women, and improving people’s general health by providing a nutritious diet. Thus, Fe, Zn, and antioxidant compounds have been prioritized for their enrichment in staple foods through biofortification [18,20,21].

Most of the traditional landraces and wild rice accessions are pigmented and these accessions constitute a significant proportion of rice gene banks. Additionally, pigmented rice cultivars, with red, purple, black, brown and yellow kernels, are still grown in some areas in Asia because of their nutritional and medicinal value or as part of traditional cultural practices [22,23,24,25]. This germplasm can be a good source of valuable genes/quantitative trait locis (QTLs) for various nutrition, grain quality, and pest and disease resistance traits for rice breeding programs [4,26,27,28,29,30,31]. Several studies on the characterization of colored rice for different antioxidant compounds, vitamins, and minerals have shown wide variations and these accessions were found to have 3- to 4-fold higher nutrient content than modern rice varieties [32,33,34,35]. Similarly, the natural variation for bacterial leaf blight (BLB) resistance in the colored rice can be used for breeding BLB resistance in rice. BLB is one of the most devastating diseases and causes significant yield losses in rice. BLB resistance is an integral part of all the breeding programs including breeding for biofortified rice varieties. Therefore, the evaluation of rice accessions for high Fe and Zn content and BLB resistance is vital in facilitating efforts to develop rice varieties with increased nutritional content.

Traditional rice varieties often possess beneficial QTLs or genes for plant improvement that are not available in current cultivated mega-varieties. Several useful QTLs in traditional varieties were identified and used by introgression or pyramiding into cultivars to improve agronomic performance [4,26,27,28,29,30,31]. Understanding the genetic basis of complex traits through the identification of major-effect QTLs/genes, and their application through maker-assisted breeding, is an attractive option to develop nutritious rice varieties. A candidate gene (*Pb*) on chromosome 4 has been identified for purple pericarp in rice [36]. In an association analysis, QTLs were detected for pericarp color on chromosomes 4 and 8 [37]. Similarly, in a panel of landraces, loci were detected for pericarp color on chromosomes 2, 7, and 8 [38]. A total of 21 QTLs were detected for proanthocyanin and anthocyanin pigments in a recombinant inbred iines RIL population [39]. Similarly, for grain Zn, several major-effect and consistent QTLs have been reported [33,40,41]. However, genome-wide association studies (GWAS), using high-density single nucleotide polymorphism (SNP) genotyping in natural populations, are an effective way to identify more precise QTLs or genes.

A diversity panel of colored rice accessions from South Korea was evaluated at International Rice Research Institute (IRRI), Philippines, and Chungcheongnam-do, South Korea. This present study was undertaken to identify QTLs for agronomic, micronutrient, and bacterial leaf blight resistance traits using a colored rice diversity panel, a shortlist of candidate genes, and the identification of promising genotypes with desirable traits.

## 2. Materials and Methods

### 2.1. Plant Materials

A diversity panel of colored rice composed of 156 accessions was evaluated in two environments, Chungcheongnam-do, South Korea, and the Robert Zeigler Experiment Station (ZES) at the IRRI, Los Baños (LB), Laguna, Philippines. The trials were laid out in a randomized complete block design (RCBD). Standard agronomic practices and plant protection measures were applied to ensure good crop growth and complete grain development.

### 2.2. Phenotyping

All the accessions were phenotyped for nine agronomic traits, days to heading (DH), number of panicles (NP), panicle length (PL), number of spikelets per panicle (NSP), ripening ratio (RR), grain length (GL); grain width (GW), thousand-grain weight (TGW), and anthocyanin (AC) in Korea, and for DH, TGW, Fe, and Zn at the IRRI. Materials were also screened for 14 *Xanthomonas oryzae* pv. *oryzae* (*Xoo*) strains for BLB resistance. All the agronomic traits were measured following the IRRI Standard Evaluation System [42]. RR is the ratio of the number of filled spikelets to the total number of spikelets. We measured Fe and Zn for all the entries and from all the replications. From each replication, 50 g of seeds were dehulled and polished for 60 s (Indo Plast), and 3 g of milled rice samples underwent X-ray fluorescence analysis using a Bruker S2 Ranger. Per sample, two readings were collected and the values were expressed in milligrams per kilogram (mg/kg). Anthocyanin content was estimated using high performance liquid chromatography (HPLC) as described in [43].

### 2.3. BLB Screening Method

The different *Xoo* strains were inoculated separately to screen for disease reactions among the 156 accessions. Leaves of each accession were clip-inoculated at maximum tillering stage. Three plants per accession per strain were inoculated with bacterial suspension containing 1 × 10^9^ CFU/mL of distilled water. Fourteen days after inoculation, the lesion length from each inoculated leaf was recorded. Lesion length was obtained by measuring the necrotic area from the point of inoculation up to the water-soaked part of the lesion. The average lesion was used for the further statistical analysis.

### 2.4. Analysis of Phenotypic Data

All the basic statistical parameters for different traits were analyzed using STAR v.2.0.1. Analysis of variance (ANOVA) and correlation estimation were carried out using PB Tools v1.4.

### 2.5. Genotypic Analysis and GWAS

DNA extraction from leaf tissues was performed using cetyl trimethyl ammonium bromide (CTAB) method. The quality and quantity of DNA was checked and normalized to 10 ng/uL. A 384-plex genotype-by-sequencing (GBS) library was prepared using the ApeKI restriction enzyme, following the protocol described in Elshire et al. (2011) [44]. The quantity and quality of the GBS library was assessed using the Bioanalyzer kit (Agilent Genomics, Santa Clara, CA, USA). Library sequencing was performed using paired-end sequencing (150 bp reads) of one 384-plex library per flow cell channel. Sequencing was performed on a HiSeq2000 (Macrogen Inc., Seoul, Korea). A total of 29,194,089 paired-end reads, each with a length of 101 base pairs (bp), were generated. The raw sequence reads were filtered based on the following criteria: (1) the sequence reads perfectly match one of the barcodes with the expected ApeKI cut site four-base remnant (CWGC), and (2) does not contain “N” within the first 64 bases after the barcode. Using the barcodes, the raw reads were processed and collapsed into sets of unique 64-bp sequence tags, which were then aligned to the Nipponbare reference genome (IRGSP 1.0) using Burrows-Wheeler Aligner (BWA) software. SNP calling was done using TASSEL-GBS plugin [45] in TASSEL version 3.0.147 [46]. SNP marker data of 157 genotypes, generated by GBS, consisting of 180,599 SNPs, were screened based on ≥80% call rate, locus homozygosity, and minor allele frequency (MAF) ≥0.05, which resulted in 22,112 SNPs. However, one accession had greater than 50% missing rate and had to be excluded from the analysis. We used phenotypic and genotypic data from 152 accessions for further analysis (Table S1). This set of SNPs was used to analyze the intra-chromosomal linkage disequilibrium (LD) (r^2^ values) between SNP marker pairs, which was calculated using TASSEL v5.2.20 with 50 LD windows. Marker pairs with statistically significant LD (*p* < 0.05) were considered in the LD analysis. An LD map was generated by plotting r^2^ values against distance (Mb) using Graphical GenoTypes (GGTPLOT2) [47]. Population structure was determined using all the SNP markers. The number of groups (K) tested varied from 1 to 10, and each K structure of the population was analyzed with three replications. Each run included a burn-in period of 10,000 steps and 10,000 Monte Carlo Markov Chain iterations. The number of groups (K) was estimated using (ΔK) [48] in Structure Harvester [48,49]. The average trait values of each accession were used for association analysis. The mixed linear model with kinship [MLM (Q+K)] approach in Genome Association Prediction Integrated Tool (GAPIT) was used to carry out GWAS. Manhattan plots were produced and a threshold value of –log (*p* value) ≥3.0 was used to declare significant marker-trait associations.

### 2.6. Candidate Gene Analysis

The physical position of peak SNP markers for each QTL was determined and used for candidate gene search. Annotated genes and gene families with their functions related to the respective traits were downloaded from Oryzabase (https://shigen.nig.ac.jp/rice/oryzabase/gene) and the physical positions of annotated genes with known functions were determined using the Rice Annotation Project – Data Base RAP DB Genome Browser (http://rapdb.dna.affrc.go.jp/viewer/gbrowse/irgsp1). Further searches were performed for previously reported QTLs that co-localized with present QTLs using the Gramene database.

## 3. Results

### 3.1. Phenotypic Analysis

**Figure 1:**
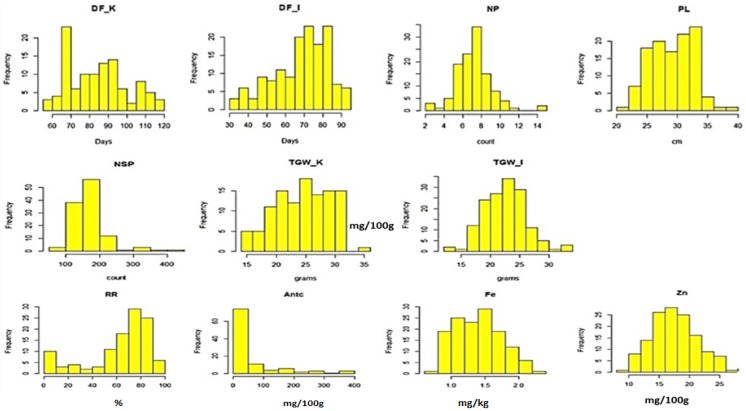
Histograms for agronomic and micronutrient traits.

The traits DH, NP, PL, TGW, Fe, and Zn were normally distributed, whereas RR and AC showed skewed distribution (Figure 1). The colored rice panel showed wide variations for all the traits. Table 1 provides the range, mean, and co-efficient of variation for different traits in the respective environments. The colored rice accessions Bir-R-Ton-Tsan and Chenlun (Hea-Li) 55 were early for DH; Akuramboda and CN09 had the highest Zn (26.5 mg/Kg); French Type and Chenlun (Hea-Li) 55 had the highest value for Fe (2.3 mg/Kg), along with Ase Pindjauand Khao Kam for AC, Akuramboda and Pulut Hitam for NP, Paro Lo’ting and Tadong1 for PL, Mah Nam Pui and Ketan Mari Kangen for NSP, DNJ51 and ARC6577 for RR, and Khao and Khao’ Bam for TGW (Table S1). Coefficients of variation (CV) for all traits varied from 12.0% to 40.2%. Three traits (DH, PL, and TGW) showed a medium CV (10–20%) and two traits (NSP and RR) showed a very high CV (>30%); whereas NP and the two micronutrient traits (Zn and Fe) had high CV (20–30%). In general, six accessions had grain Zn of 25 mg/Kg or more, ten accessions had grain Fe of more than 2 mg/Kg, and eight accessions had more than 250 mg/100 g for anthocyanin content. The anthocyanin was in the range of 94.70–202.46 mg Cy-3-glc/100 g db in Indonesian pigmented rice [50].

**Figure 2:**
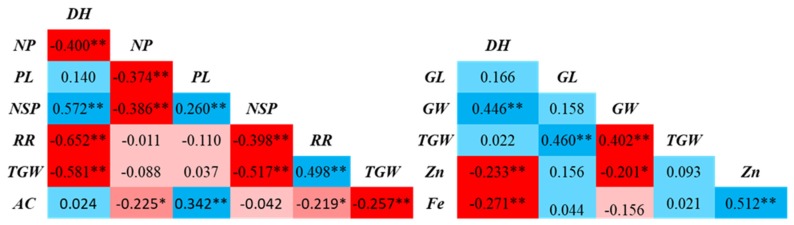
Correlation among different traits evaluated at two different locations.

The accessions showed wide variations in response to 14 different *Xoo* strains. The disease score measured as lesion length (LL) showed skewed distributions. The lesion length in response to *Xoo* strains PXO61 and PXO339 showed the widest and narrowest LL variation, respectively. Figure 2 shows the correlation among the traits. Of the 36 possible correlations among all traits evaluated in both environments, a total of 17 were significant; three of them were positively correlated and 11 were negatively correlated. DH was negatively correlated with NP, RR, and TGW, but positively correlated with NSP; NP was negatively correlated with PL, NSP, and AC; PL was positively associated with NSP and AC; NSP was negatively correlated with RR and TGW; RR was positively associated with TGW, but negatively with AC; and TGW was negatively associated with AC. Similarly, Fe and Zn were negatively correlated with DH and Fe, and Zn had a highly significant positive correlation.

### 3.2. Genetic Analysis and Linkage Mapping

**Figure 3:**
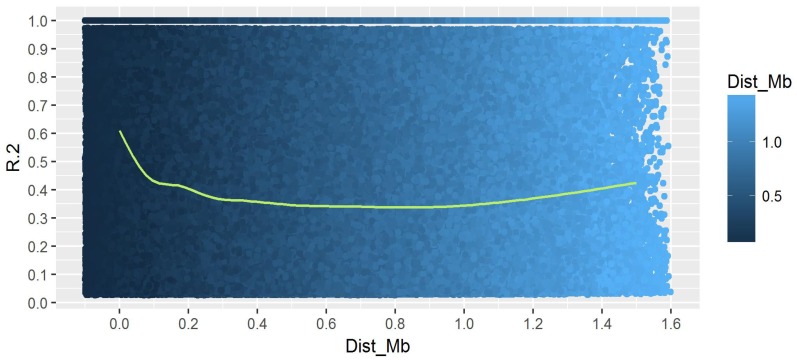
LD decay in the colored rice panel using 22,112 markers.

GBS data were aligned with the reference genome Nipponbare and SNPs were called. A total of 22,112 SNP markers (with average density of 19.4 kb) covering all 12 chromosomes were used to access the genetic structure of the 152 rice accessions. The number of SNPs on each chromosome varied from 1219 on chromosome 12 to 3315 on chromosome 1. All the other chromosomes had more than 1400 SNPs each. The magnitude of LD and its decay with genetic distance determine the resolution of association mapping. The LD analysis in the colored rice panel identified 809,803 pairs (74.3%) in complete LD (Table 2). The shortest physical distance group (0–5 kb) had the highest average LD (0.653). The decay declined to 0.340 average LD (48% decline) at a physical distance of >750 to 1000 bp, before it increased at a distance of more than 1000 kb (Figure 3). In addition, the same pattern was observed for the percentage of marker pairs in complete LD, for which it decreased from 36.5%, in the shortest distance group, to 1.7% at a physical distance of >750 to 1000 bp.

### 3.3. Structure and Principal Component Analysis

**Figure 4:**
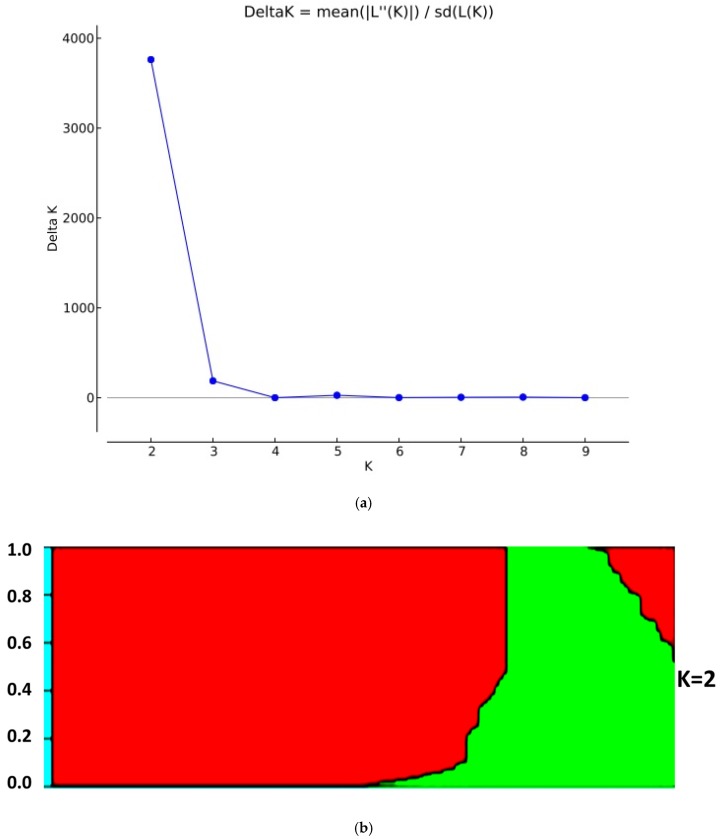
(**a**) Population structure in the colored rice panel. (**b**) Bar plot representing the cluster membership of each accession detected by structure.

**Figure 5:**
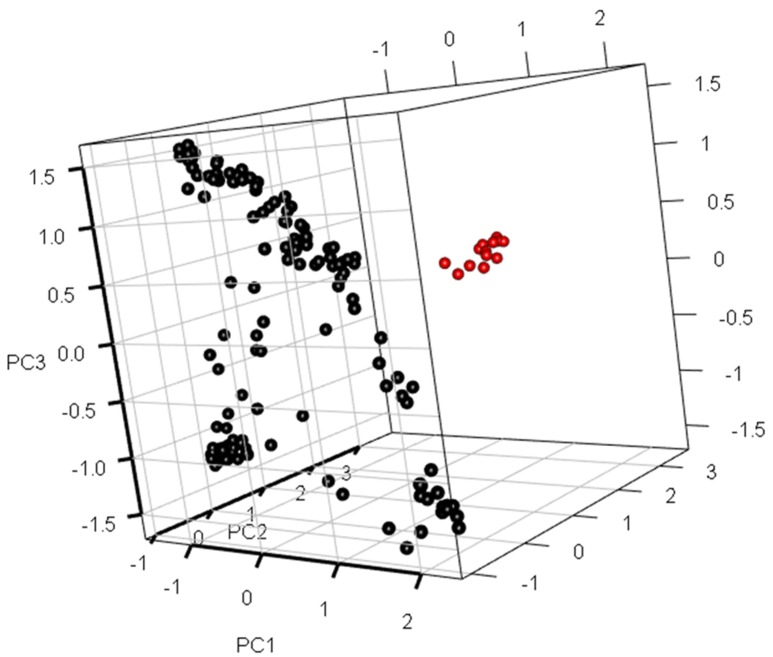
3D scatter plot of the first three principal component analysis (PCA).

Population structure analysis, using a subset of SNP markers, revealed that the variance of log likelihood increased from K = 1 to K = 10, and the highest ΔK of 3762.2 was observed at K = 2 (Figure 4a,b), indicating that the population can be divided into two subgroups with 114 and 42 individuals belonging to clusters 1 and 2, respectively. The cluster memberships of each individual (Q1) and kinship data for all traits were used for GWAS. These varieties were assigned to three genetic clusters in a three-dimensional plot of the first three principal components (i.e., PC1, PC2, and PC3). Using a three-dimensional (3D) scatter plot of principal component analysis (PCA), and based on 22,121 SNPs, two major clusters were clearly distinguished among all the colored rice accessions (Figure 5), which is consistent with results from population structure analysis as it also grouped the accessions into two subgroups. Rice genotypes from cluster 1 were depicted by red color, whereas cluster 2 genotypes were represented by black color. The first three PCs accounted for 45% of the total variance breakdown of this cumulative variance value, which revealed contributions of 35%, 6%, and 4% for PC1, PC2, and PC3, respectively.

### 3.4. Genome-Wide Association Mapping

**Figure 6:**
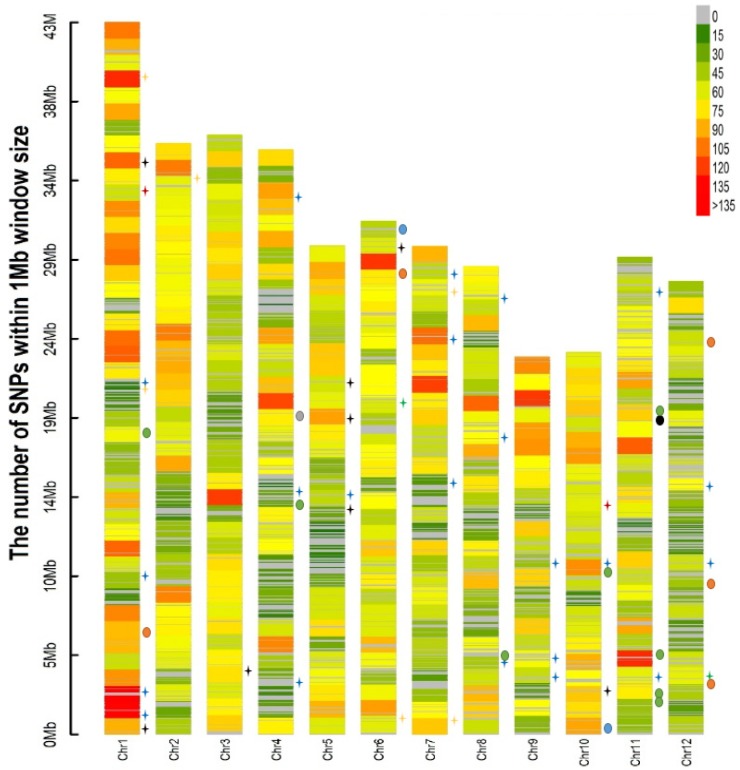
Single nucleotide polymorphism (SNP)-density plot and QTL positions for agronomic traits, nutrition, and bacterial leaf blight (BLB) resistance. 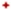: Antc; 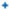: BLB; 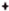: DH; 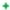: Fe; 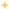: GW; 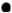: NP; 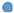: PL; 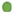: RR; 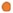: Zn; and 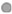: GL.

In total, 35 QTLs were identified for agronomic and nutritional traits at *p* < 0.0001 (Table 3 and Figure 6). They were distributed on all the chromosomes, except on chromosome 7, and the phenotypic variance explained (PVE) of these QTLs varied from 9% to 18%. The highest number of QTLs was identified on chromosome 1 (seven), followed by chromosomes 6 and 11, each with five QTLs. On other chromosomes, the number of QTLs varied from one to four. Similarly, for BLB resistance, 22 QTLs were identified on chromosomes 1, 4, 7, 8, 9, 10, 11, and 12. The highest number of QTLs was identified on chromosome 1 (four), followed by chromosomes 4, 7, 8, and 9, each with three QTLs. The PVE of the QTLs varied from 9.9% to 12.9%. Four QTLs were identified for resistance to POX330, and three QTLs each for resistance to PXO61 and PXO99. The probability of false detection rate (FDR) estimation was non-significant for all the QTLs. Details of the QTLs identified for BLB resistance traits are provided in Table 4.

### 3.5. QTLs for Agronomic and Nutritional Traits

1. DH: Eight QTLs were identified for days to heading on chromosomes 1, 3, 5, 6, and 10. Most of them were located on chromosome 5 (Table 3). The PVE of these QTLs varied from 9.3% to 14.7%. *qDH_1.1_*, *qDH_1.3_*, *qDH_5.2_*, *qDH_6.1_*, and *qDH_10.1_* had PVE of more than 10% each. *qDH_5.2_* had the highest PVE (14.7%).
2. GL: Only one QTL (*qGL_4.1_*) was identified for grain length on chromosome 4. The PVE of this QTL was 14%.
3. GW: Two QTLs were identified for grain width, one each on chromosomes 2 and 4. *qGW_2.1_* and *qGW_6.1_* had a PVE of 12.6% and 14.4%, respectively; both were identified at a very high *p*-value.
4. NP: Only one QTL (*qNP_1.1_*) was identified on chromosome 11, with a PVE of 16.7%.
5. PL: For panicle length, two QTLs were identified on chromosomes 6 (*qPL_6.1_*) and 10 (*qPL_10.1_*), with a PVE of 12.9% and 12.6%, respectively.
6. RR: In all, eight QTLs were identified for ripening ratio on chromosomes 1, 4, 8, 10, and 11. Most of them were located on chromosome 11. The PVE of these QTLs varied from 12.0% to 17.5%. The QTL *qRR_11.4_* had the highest PVE (17.5%).
7. TGW: Four QTLs were identified for grain weight, two each on chromosomes 1 and 7. Three (*qTGW1.1*, *qTGW_1.2_*, and *qTGW_7.1_*) of them had a PVE of more than 10%.
8. Fe: Two QTLs were identified for grain Fe content, one each on chromosomes 6 and 12. Both had a PVE of more than 10%.
9. Zn: Five QTLs were identified for grain Zn content on chromosomes 1, 6, and 12, with three of them on chromosome 12. All of them had a PVE of more than 10%; *qZn_12.2_* had the highest PVE (17.9%).
10. AC: Two QTLs were identified for anthocyanin content, one each on chromosomes 1 and 10, with a PVE of 14.5% and 13.1%, respectively.

### 3.6. QTLs for BLB Resistance

A total of 22 QTLs were mapped only for 11 of the 14 different *Xoo* strains screened on the colored rice panel. All of the QTLs had a PVE of more than 10%, except *qBLB_1.2_*, *qBLB_1.4_*, and *qBLB_4.3_* (Table 4 and Figure 6). Four QTLs (*qBLB_4.3_*, *qBLB_5.1_*, *qBLB_7.2_*, and *qBLB_7.3_*) were identified for resistance to *Xoo* strain PXO363. Three QTLs each were identified for resistance to *Xoo* strains PXO61 and PXO99.

### 3.7. Candidate Genes Co-Located with the QTLs

Most of the QTLs detected were co-located with either known QTLs or genes for the respective traits; also, some new QTLs were identified (Table 3). The PVE of the QTLs varied from 9.3% to 17.9% (Table 3). The Antc QTL *qAntc_1.1_* was co-located with the *glu4* gene. The DH QTLs were co-located with *PME1, lsi2, siz1, OsLti6b, dth1.1*, *OsCrRLK1L2*, and *OsFD2***.** *qGL_4.1_* co-located with *GIF1*. Similarly, grain weight QTLs *qGW_1.2_*, *qGW_6.1_*, and *qGW_7.1_* were co-located with *OsaLeg1*, *SSG6*, *qgw1.1*, *qgrl1-1*, *AQED046*, and *SDGP7* (Table 3). For Fe and Zn, three of the seven QTLs were co-located with the metal homeostasis genes (Table 3). For BLB resistance, eight of the 22 QTLs (*qBLB_1.4_*, *qBLB_4.3_*, *qBLB_5.1_*, *qBLB_7.1_*, *qBLB_7.3_*, *qBLB_8.1_*, *qBLB_8.3_*, and *qBLB_11.2_*) were co-located with 12 known BLB genes or with their functionally-related genes *(OsLOL2, Xa1, OsWRKY45, xa5, xa8, oscbt, rtGA2.1, OsGLP8, Os8N3, xa13, Xa3*, and *Xa26*) (Table 4). The PVE of the QTLs varied from 9.9% to 12.9% (Table 4).

### 3.8. Identification of Donor Lines for Grain Zn and Anthocyanin

Among the colored rice samples, six high-Zn lines and another six high-AC lines were identified and are presented in Table 5. Among the selected lines, Zn content ranged from 24.8 to 26.6 mg/kg, whereas AC was more than 295.6 mg/Kg. However, only one accession, namely, Sayllebon, contained higher content of both Zn (24.8 mg/kg) and AC (375.4 mg/kg).

## 4. Discussion

Colored rice is a rich source of vitamins and minerals, containing several-fold higher nutrients than regularly consumed white rice, so it can contribute significantly to human health and nutrition [94,95,96]. Thus, efforts are being made to conserve, characterize, and cultivate colored rice accessions and promote the consumption of colored rice and brown rice as part of the major health and nutrition initiatives in many countries of Asia [24,97]. In the present study, we characterized colored rice accessions for nutritional, agronomic, and BLB resistance traits, and carried out association mapping and candidate gene analysis to facilitate the development of healthier rice varieties.

Biofortification has been proven to be one of the most cost-effective methods in combating Fe and Zn deficiencies [40,98,99,100,101,102]. However, the accumulation of mineral elements in the edible parts is a complex process involving multiple QTLs/genes and is highly influenced by environmental factors [33,103]. Biofortified rice varieties, with high grain mineral concentration, should be high yielding, with desirable grain quality traits, and resistant to major pests and diseases for their successful adoption [16,104]. Thus, an understanding of the molecular basis of all of these complex traits will help in precisely pyramiding several genes and QTLs to develop superior and farmer-adoptive nutritious rice varieties.

Mostly normal frequency distribution was observed for all the nutritional and agronomic traits, and skewed distribution for BLB resistance, indicating their polygenic and oligogenic/monogenic genetic control, respectively, which is the normal trend reported for these traits [105,106,107,108]. Some of the accessions with higher content of Zn and AC were CR0021, Quakor and Trunia and Sayllebon, Filiwa and Koni, respectively. They are useful for breeding as well as to directly promote them as healthier rice. Selection of rice donor parents with multiple beneficial traits seems to be a good strategy to reduce the impact of linkage drag in breeding. Our study found that red rice genotype, Sayllebon, which is high in both Zn and AC, could be a valuable material for a breeding program for nutritious rice. Sayllebon/3-203 was also found to contain a higher amount of antioxidant compound γ-oryzanol (9.1 mg/100 g hulled rice) [109]. Even though higher Fe, Zn, and AC were reported in colored rice, our results showed that there was not much variation for Fe, but wide ranges were observed for Zn (9.2 to 26.6 mg/Kg) and AC [2,35,107,110,111,112]. A wide range of variability for different traits indicated the role of genotype as well as environmental effects on the expression of these traits. In general, rice accessions have less variability for Fe in the endosperm. Some of the colored rice genotypes may have high Fe in the endosperm; such accessions may be very rare. In contrast, the huge variability available for grain Zn, anthocyanin content, and antioxidant compounds in colored rice can be exploited in breeding programs.

The correlations among nutritional and agronomic traits exhibited known trends. DH was negatively correlated with yield components, such as NP, RR, and TGW, and positively correlated with NSP; TGW was negatively associated with AC but, interestingly, it did not show any significant relationship with Fe and Zn; AC was negatively correlated with most of the yield components and significantly positively correlated only with PL. Similarly, Fe and Zn were negatively correlated with DH, whereas Fe and Zn had a highly significant positive correlation. Number of spikelets per panicle (NSP) is an important trait to determine the number of grains per unit area. Large variation in NSP was reported in previous studies [113,114,115]. According to Kato (1986) [116], RR is usually low in rice genotypes with a higher number of grains, which is in agreement with our results. Further, the significant positive correlation identified between Fe and Zn was supported by earlier findings [117,118]. Fe and Zn share the same genomic region or genes or biochemical pathways [119,120]. Even though QTLs/genes for highly correlated traits are co-located, they may be tightly linked or pleiotropic, but linkage drag must be eliminated through pre-breeding or precise introgression of the target region in elite genetic backgrounds [121].

The SNP density used in the analysis was very high, with an average density of one SNP for every 19.4 kb. All the chromosomal regions were well covered without any significant gaps. A high-density SNP is desired for accurate QTL/gene detection [122,123]. In our population, limited subgroups and a higher number of marker pairs in complete LD were detected. The structure and PC analyses clearly grouped the accessions into two subgroups without admixtures, indicating free flow of genes within groups and less gene pool sharing across groups. We detected 74.3% of the marker pairs in complete LD, with an average LD distance of 1000 kb, and, interestingly, one-third (36%) of the LD pairs were less than 5 Kb, indicating a high rate of recombination in the panel. The identification of subgroups and generating kinship data for use in association analysis are important to avoid spurious associations [124,125]. The low average LD distance with high recombination is essential for any association analyses to accurately detect the precise location of QTLs/genes [122]. We used structure with kinship (Q+K) information for GWAS analyses.

Genome-wide association study is widely used for genetic analyses to exploit genetic diversity in rice and to identify novel alleles for multiple traits [119,126,127,128,129,130]. In our study, GWAS detected multiple major loci for all the traits except for NSP and TGW. In total, 35 QTLs were identified for nutritional and agronomic traits at *p* < 0.0001 (Table 4). They were distributed on all the chromosomes, except on chromosome 7, and the PVE of these QTLs varied from 9% to 18%. The highest numbers of QTLs were identified for DH and RR. It is also interesting to note that 18 (51%) QTLs for agronomic and micronutrient traits were co-located with either known QTLs or genes for the respective traits. Previous studies reported several QTLs and genes for agronomic, yield, and nutritional traits in various biparental and natural mapping populations in rice [119,131,132,133,134,135,136,137]. For BLB resistance, 22 QTLs were identified; 12 (54%) of them were co-located with known BLB genes or with their functionally-related genes. The PVE of the QTLs varied from 9.9% to 12.9% (Table 4). The co-locations of candidate genes with QTLs provide the evidence for accuracy in mapping of genetic loci. With the recent advancements in rice genomics, there has been increasing accumulation of information on QTLs and genes for various traits in rice using different approaches [138,139]. Some of the genes for disease resistance, nutritional and grain quality, and agronomic traits have been cloned, functionally validated, and successfully used in breeding programs [139,140]. However, there is a need to continuously search for novel alleles to cater to the diverse needs of breeding programs to mitigate the adverse effects of climate change and to meet the food and nutritional demands of farmers and consumers [38].

Prioritization of the candidate genes underlying major-effect QTLs for complex traits, and their functional validation, is necessary to understand their influence on phenotype and also to develop functional markers for use in breeding [141,142,143,144]. Several QTLs identified in this study were co-located with known or functionally-related genes. *qAntc_1.1_* was co-located with the *glu4* gene, which is related to seed glutelin quality (eating quality). The DH QTLs were co-located with *PME1, lsi2, siz1, OsLti6b, dth1.1*, *OsCrRLK1L2ˆ,* and *OsFD2*. *PME1, siz1ˆ,* and *OsFD2* genes are known to be involved in anther and leaf development [53,55,57]. *qGL_4.1_* co-located with *GIF1,* the gene that regulates grain size and grain filing in rice [59]. Similarly, grain weight QTLs *qGW_1.2_*, *qGW_6.1_*, and *qGW_7.1_* were co-located with *OsaLeg1*, *SSG6*, *qgw1.1*, *qgrl1-1*, *AQED046*, and *SDGP7. SSG6* is a substandard starch granule-6 gene that is involved in the development of large starch granules in the endosperm [61], whereas *AQED46* is related to 1000-grain weight [62]. The results of this study suggest that SSG6 is a novel protein that controls grain size. SSG6 will be a useful molecular tool for future starch breeding and applications. For Fe and Zn, three of the seven QTLs were co-located with the metal homeostasis genes. These were zinc ion transporter, *OsCNGC16*, *OsDof*, *OsHMA9*, *OsNRAMP3*, *OsbZip85*, *OsNRAMP7*, and *ZFP252*.

Among the BLB resistance QTLs, eight of the 22 QTLs (*qBLB_1.4_, qBLB_4.3_, qBLB_5.1_, qBLB_7.1_, qBLB_7.3_, qBLB_8.1_, qBLB_8.3_,* and *qBLB_11.2_*) were co-located with 12 known BLB genes or with their functionally-related genes *(OsLOL2, Xa1, OsWRKY45, xa5, xa8, oscbt, rtGA2.1, OsGLP8, Os8N3, xa13, Xa3*, and *Xa26*), respectively. Furthermore, the BLB resistance gene-rich region of chromosome 8 was identified with two QTLs (*qBLB_8.1_* and *qBLB_8.3_)* in which *OsGLP8, Os8N3*, and *xa13* were found to be co-located. *qBLB_11.2_* also co-located with two important BLB genes, *Xa3* and *Xa26.* This *qBLB_11.2_* was also identified by other studies [145,146,147,148,149]. GWAS also identified SNP markers near known genes that are related to disease resistance and stress tolerance such as *OsRLCK19*, *OsLOL2*, and *OsGLP8*, which are plausible candidate resistance genes on chromosomes 1 and 8. Similarly, a GWAS in a ulti-Parent Advanced Generation Inter-Cross (MAGIC) population identified several major and minor loci for BLB resistance [150]. Although major BLB resistance genes have been successfully introgressed using marker-assisted breeding, widespread use of these varieties narrows the genetic base, resulting in high selection pressure against the prevailing BLB strains, resulting in more virulent strains that could overcome the major R genes of rice. Thus, pyramiding of small-effect BLB QTLs along major genes is necessary for a more sustainable BLB resistance [106,151]. This *qBLB_11.2_* region was also very close to the location of four reported blast resistance genes, namely, *Pik1*, *Pik2*, *Pikm*, and *Pik-p* [152,153,154]; thus, *qBLB_11.2_* might be useful for the development of BLB and blast disease resistance in rice.

## 5. Conclusions

This study reports QTLs identified for nutritional, agronomic, and BLB resistance traits using GWAS in a colored rice diversity panel. Colored rice accessions have higher nutritive value than non-pigmented rice genotypes as they contain higher levels of micronutrients and antioxidant compounds. The lines with higher nutritional value identified in this study could be used as donor parents for nutritional traits, and crossed with high-yielding mega-varieties to develop new high-yielding and nutritious rice cultivars. Furthermore, the identified BLB resistance loci might be beneficial in developing rice cultivars resistant to BLB. The introgression of major-effect QTLs for the desired traits identified in this study will also enhance the efficiency of marker-assisted breeding programs.

## Figures and Tables

**Table 1:** Descriptive statistics of agronomic and micronutrient traits in colored rice.

Trait	Range	Mean ± SE	CV (%)	Location
DH	58–119	84.51 ± 1.49	18.2	K
	34–94	68.94 ± 1.21	21.2	I
NP	2–15	7.47 ± 0.18	26.3	K
PL	21.5–40.0	29.39 ± 0.33	12.0	K
NSP	70.5–406.0	171.73 ± 4.86	30.3	K
RR	0.2–93.6	63.32 ± 2.42	40.2	K
GL	0.48–0.75	0.63 ± 0.0056	9.03	K
GW	0.21–0.33	0.26 ± 0.0025	9.75	K
TGW	14.2–34.6	24.62 ± 0.44	18.7	K
	13.4–32.9	22.51 ± 0.29	15.8	I
AC	0.8–375.4	57.33 ± 8.92	-	K
Zn	9.2‒26.6	17.56 ± 0.31	20.7	I
Fe	0.7‒2.3	1.41 ± 0.03	25.5	I

DH = days to heading; PH = plant height (cm), NP = number of panicles; PL = panicle length (cm); NSP = number of spikelets per panicle; RR = ripening ratio; GL= grain length; GW = grain width; TGW = thousand-grain weight (g); AC = anthocyanin content (mg/100 g); Zn = zinc (mg/kg); Fe = iron (mg/kg); K = Korea; I = IRRI, Philippines.

**Table 2:** Linkage disequilibrium (LD) in the colored rice panel.

Distance (kb)	Average LD (*p* < 0.05) r^2^	Significant LD Pairs	Marker Pairs in LD	Marker Pairs in LD (%) per Distance Group
0–5	0.653	19,394	7077	36.49
>5–100	0.491	117,154	12,783	10.91
>100–250	0.409	164,039	7264	4.43
>250–500	0.361	242,032	5408	2.23
>500–750	0.341	160,178	2531	1.58
>750–1000	0.340	63,238	1133	1.79
>1000	0.383	43,048	2151	5.00
Total	-	809,803	38,347	-

**Table 3:** Quantitative trait loci (QTL) for agronomic and nutritional traits.

Trait	QTL	Peak Marker	Chr	Position (bp)	*p*-Value	R^2^ (%)	QTL	Reference
Antc	*qAntc_1.1_*	S1_33820736	1	33,820,736	3.64 × 10^−4^	14.5	*glu4a*	[51]
Antc	*qAntc_10.1_*	S10_13377773	10	13,377,773	4.33 × 10^−4^	13.1	*PLA1*	[52]
DH	*qDH_1.1_*	S1_729480	1	729,480	3.39 × 10^−4^	13.3	-	-
DH	*qDH_1.2_*	S1_35193967	1	35,193,967	4.06 × 10^−4^	9.4	*PME1*	[53]
DH	*qDH_3.1_*	S3_4363021	3	4,363,021	3.11 × 10^−4^	14.7	*lsi2*	[54]
DH	*qDH_5.1_*	S5_14036335	5	14,036,335	5.00 × 10^−4^	9.8	*siz1*	[55]
DH	*qDH_5.2_*	S5_19759126	5	19,759,126	4.14 × 10^−4^	10.3	-	-
DH	*qDH_5.3_*	S5_21908094	5	21,908,094	4.88 × 10^−4^	9.3	*OsLti6b*	[56]
DH	*qDH_6.1_*	S6_30059999	6	30,059,999	3.63 × 10^−4^	10.9	*OsFD2*	[57]
DH	*qDH_10.1_*	S10_3764176	10	3,764,176	3.00 × 10^−4^	11.1	-	-
Fe	*qFe_6.1_*	S6_21751078	6	21,751,078	5.39 × 10^−4^	10.3	*qFe6*	[58]
Fe	*qFe_12.1_*	S12_3936500	12	3,936,500	5.58 × 10^−4^	10.6	-	-
GL	*qGL_4.1_*	S4_20409894	4	20,409,894	4.79 × 10^−4^	14.0	*GIF1*	[59]
GW	*qGW_1.1_*	S1_22901457	1	22,901,457	4.28 × 10^−4^	13.3	*OsaLeg1*	[60]
GW	*qGW_1.2_*	S1_40478067	1	40,478,067	4.06 × 10^−4^	12.6	*-*	-
GW	*qGW_2.1_*	S2_34634978	2	34,634,978	7.54 × 10^−4^	12.6	*-*	*-*
GW	*qGW_6.1_*	S6_1344933	6	1,344,933	5.10 × 10^−4^	14.4	*SSG6*	[61]
GW	*qGW_7.1_*	S7_1189467	7	1,189,467	3.41 × 10^−4^	9.8	*AQED046*	[62]
GW	*qGW_7.2_*	S7_27630784	7	27,630,784	3.47 × 10^−4^	12.5	*-*	-
NP	*qNP_11.1_*	S11_21353461	11	21,353,461	8.86 × 10^−5^	16.7	*-*	-
PL	*qPL_6.1_*	S6_30785431	6	30,785,431	5.32 × 10^−4^	12.9	*OsSSI*	[63]
PL	*qPL_10.1_*	S10_94178	10	94,178	5.32 × 10^−4^	12.6	*-*	-
RR	*qRR_1.1_*	S1_18464561	1	18,464,561	5.02 × 10^−4^	12.0	*-*	-
RR	*qRR_4.1_*	S4_13845093	4	13,845,093	3.97 × 10^−4^	14.5	*RERJ1*	[64]
RR	*qRR_8.1_*	S8_5478727	8	5,478,727	3.15 × 10^−4^	14.2	*-*	-
RR	*qRR_10.1_*	S10_12185948	10	12,185,948	5.02 × 10^−4^	13.2	*-*	-
RR	*qRR_11.1_*	S11_2918255	11	2,918,255	4.85 × 10^−4^	14.0	*-*	-
RR	*qRR_11.2_*	S11_3031310	11	3,031,310	5.51 × 10^−4^	13.6	*-*	-
RR	*qRR_11.3_*	S11_6596315	11	6,596,315	3.45 × 10^−4^	15.3	*OsCIPK15*	[65]
RR	*qRR_11.4_*	S11_24271121	11	24,271,121	9.61 × 10^−5^	17.5	*RCN1*	[66]
Zn	*qZn_1.1_*	S1_6783213	1	6,783,213	3.18 × 10^−4^	11.9	*-*	-
Zn	*qZn_6.1_*	S6_28504959	6	28,504,959	8.65 × 10^−5^	15.3	*OsHMA9, OsMAPK6*	[67,68]
Zn	*qZn_12.1_*	S12_5125546	12	5,125,546	3.88 × 10^−4^	12.7	*-*	-
Zn	*qZn_12.2_*	S12_9550577	12	9,550,577	9.05 × 10^−5^	17.9	*-*	-
Zn	*qZn_12.3_*	S12_24162402	12	24,162,402	2.90 × 10^−4^	12.4	*OsNRAMP7, mel2, ZFP252*	[69,70,71]

**Table 4:** Genomic regions associated with leaf blight resistance.

*Xoo* Strain	QTL	Peak Marker	Chr	Position (bp)	*p*-Value	R^2^ (%)	Genes	Reference
PXO145	*qBLB_1.1_*	S1_1500065	1	1,500,065	4.77 × 10^−4^	10.2	*RLCK19/OsRLCK19*	[72]
PXO61	*qBLB_1.2_*	S1_2592129	1	2,592,129	5.09 × 10^−4^	9.9	*-*	-
PXO280	*qBLB_1.3_*	S1_10892915	1	10,892,915	5.20 × 10^−4^	11.4	*-*	-
PXO341	*qBLB_1.4_*	S1_22881113	1	22,881,113	5.40 × 10^−4^	9.9	*OsLOL2*	[73]
PXO347	*qBLB_4.1_*	S4_3958984	4	3,958,984	5.61 × 10^−4^	12.0	*-*	-
PXO280	*qBLB_4.2_*	S4_14640816	4	14,640,816	4.49 × 10^−4^	11.4	*-*	-
PXO363	*qBLB_4.3_*	S4_33881790	4	33,881,790	5.55 × 10^−4^	9.9	*OsLG1, Xa1*	[74,75,76,77,78,79,80]
PXO363	*qBLB_5.1_*	S5_14178355	5	14,178,355	5.58 × 10^−4^	12.7	*OsWRKY45, xa5*	[76,77]
PXO363	*qBLB_7.1_*	S7_15511270	7	15,511,270	4.55 × 10^−4^	10.5	*OsPHR2, Rurm1, oscbt, xa8*	[81,82,83,84]
PXO341	*qBLB_7.2_*	S7_24896976	7	24,896,976	4.32 × 10^−4^	11.3	*-*	-
PXO363	*qBLB_7.3_*	S7_29012591	7	29,012,591	4.76 × 10^−4^	11.8	*OsRLCK241, rtGA2.1, fzp*	[72,85,86]
PXO339	*qBLB_8.1_*	S8_5260173	8	5,260,173	5.20 × 10^−4^	11.7	*OsGLP8*	[87]
PXO339	*qBLB_8.2_*	S8_17538523	8	17,538,523	4.86 × 10^−4^	10.6	*-*	-
PXO112	*qBLB_8.3_*	S8_26167157	8	26,167,157	4.38 × 10^−4^	10.6	*xa13, Os8N3*	[88,89]
PXO99	*qBLB_9.1_*	S9_4075062	9	4,075,062	4.12 × 10^−4^	11.2	*-*	-
PXO61	*qBLB_9.2_*	S9_5308616	9	5,308,616	5.22 × 10^−4^	10.8	*-*	-
PXO112	*qBLB_9.3_*	S9_10899580	9	10,899,580	4.57 × 10^−4^	12.9	*-*	-
PXO99	*qBLB_10.1_*	S10_11388943	10	11,388,943	4.49 × 10^−4^	10.4	*-*	-
PXO79	*qBLB_11.1_*	S11_4583814	11	4,583,814	4.60 × 10^−4^	10.4	*OsRLCK315*	[72]
PXO340	*qBLB_11.2_*	S11_27999035	11	27,999,035	4.88 × 10^−4^	10.4	*Xa3 and Xa26*	[90]
PXO61	*qBLB_12.1_*	S12_11555404	12	11,555,404	4.94 × 10^−4^	10.7	*OsRBCS3*	[91]
PXO99	*qBLB_12.2_*	S12_15461931	12	15,461,931	4.96 × 10^−4^	10.9	*PSTOL1, OsORC3*	[92,93]

**Table 5:** Colored rice accessions with high grain Zn and anthocyanin.

Germplasm	Zn (mg/kg)	Germplasm	AC (mg/kg)
CR0021	26.6	Sayllebon	375.4
Pantia	25.6	Filiwa	368.3
Quakor	25.1	Koni	361.6
Trunia	25.0	Fosagbe	303.3
Partio	24.9	Khao	296.4
Sayllebon	24.8	Ketan Kuwule	295.6

## Supplementary Materials

The following will be available online at http://www.mdpi.com/2073-4425/10/1/30/s1, Table S1: List of accessions used in the GWAS analysis.
